# Hydrogen Water: Extra Healthy or a Hoax?—A Systematic Review

**DOI:** 10.3390/ijms25020973

**Published:** 2024-01-12

**Authors:** Gagandeep Dhillon, Venkata Buddhavarapu, Harpreet Grewal, Pranjal Sharma, Ram Kishun Verma, Ripudaman Munjal, Ramprakash Devadoss, Rahul Kashyap

**Affiliations:** 1 Department of Internal Medicine, University of Maryland Baltimore Washington Medical Center, Glen Burnie, MD 21061, USA; 2 Banner Medical Group, Banner Health, Phoenix, AZ 85206, USA; venkatabuddhavarapu@gmail.com; 3 Department of Radiology, Florida State University School of Medicine, Pensacola, FL 32514, USA; harpreetsinghgrewal@gmail.com; 4 Department of Internal Medicine, Northeast Ohio Medical University, Rootstown, OH 44272, USA; sharma.pranjalmd@gmail.com; 5 Department of Sleep Medicine, Parkview Health System, Fort Wayne, IN 46845, USA; vermark2006@gmail.com; 6 Department of Nephrology, Touro University College of Osteopathic Medicine, Vallejo, CA 94592, USA; ripudaman.munjal@gmail.com; 7 Interventional Cardiology, Carle Methodist Medical Center, Peoria, IL 61636, USA; ramprakash2000@gmail.com; 8 Department of Research, WellSpan Health, York, PA 17403, USA; kashyapmd@gmail.com

**Keywords:** hydrogen water, hydrogenated water, hydrogen-rich water, antioxidant, anti-apoptotic, anti-inflammatory

## Abstract

Hydrogen-rich water (HRW) has emerged as a novel approach in the field of health and wellness. It is believed to have therapeutic antioxidant properties that can neutralize harmful free radicals in the human body. It has also been shown to be beneficial in mitigating oxidative stress-induced damage through its anti-inflammatory and anti-apoptotic pathways. We aim to conduct a systematic review to evaluate the potential benefits of hydrogen-rich water. The review protocol was uploaded on PROSPERO. After the initial search criteria, the articles were reviewed by two blinded investigators, and a total of 25 articles were included in the systematic review. The potential benefits of hydrogen-rich water on various aspects of health, including exercise capacity, physical endurance, liver function, cardiovascular disease, mental health, COVID-19, oxidative stress, and anti-aging research, are a subject of growing interest and ongoing research. Although preliminary results in clinical trials and studies are encouraging, further research with larger sample sizes and rigorous methodologies is needed to substantiate these findings. Current research needs to fully explain the mechanisms behind the potential benefits of hydrogen-rich water. Continued scientific exploration will provide valuable insights into the potential of hydrogen-rich water as an adjunctive therapeutic approach in the future.

## 1. Introduction

Hydrogen water, also known as hydrogen-rich water or hydrogenated water, is regular water that has molecular hydrogen gas (H_2_) added to it [1]. Water can be hydrogenated by dissolving molecular hydrogen gas into water under elevated pressure, resulting in a supersaturated solution. The hydrogen molecules are extremely small, so they can easily penetrate water and stay dissolved for a while [1]. Hydrogen-rich water has recently gained significant attention as a potential health-promoting beverage. Studies have been done on animals [2] and humans [3] in the last few decades using molecular hydrogen-enhanced water showing antioxidant [3], anti-inflammatory [3], and anti-apoptotic [3] effects. Although there has been some research into the benefits of hydrogen-rich water, there is still a long way to go.

Over the last few years, hydrogen-rich water has become the latest trend to target the global market in the health and wellness industry. Studies have been undertaken to understand its potential benefits. A randomized, double-blind, controlled trial [3] showed that hydrogen-rich water could reduce inflammatory responses in adults, leading to increased antioxidant capacity in healthy adults. Healthy adults consumed either 1.5 L/day of hydrogen-rich water or plain water. Flow cytometry testing of CD4+, CD8+, CD11+, CD 14+, and CD 20+ yielded interesting results. In the hydrogen-rich water group, the CD14+ cell frequency was decreased [3]. The benefits of hydrogen use have been evaluated in conditions such as cardiac fibrosis, neuronal disease, hepatic injury, radiation-induced disease, diabetes, and many more conditions [4]. Through this systematic review, we aim to summarize current research findings related to the use of molecular hydrogen-enhanced water and its anti-inflammatory, antioxidant, and anti-apoptotic impact.

## 2. Materials and Methods

**Figure 1:**
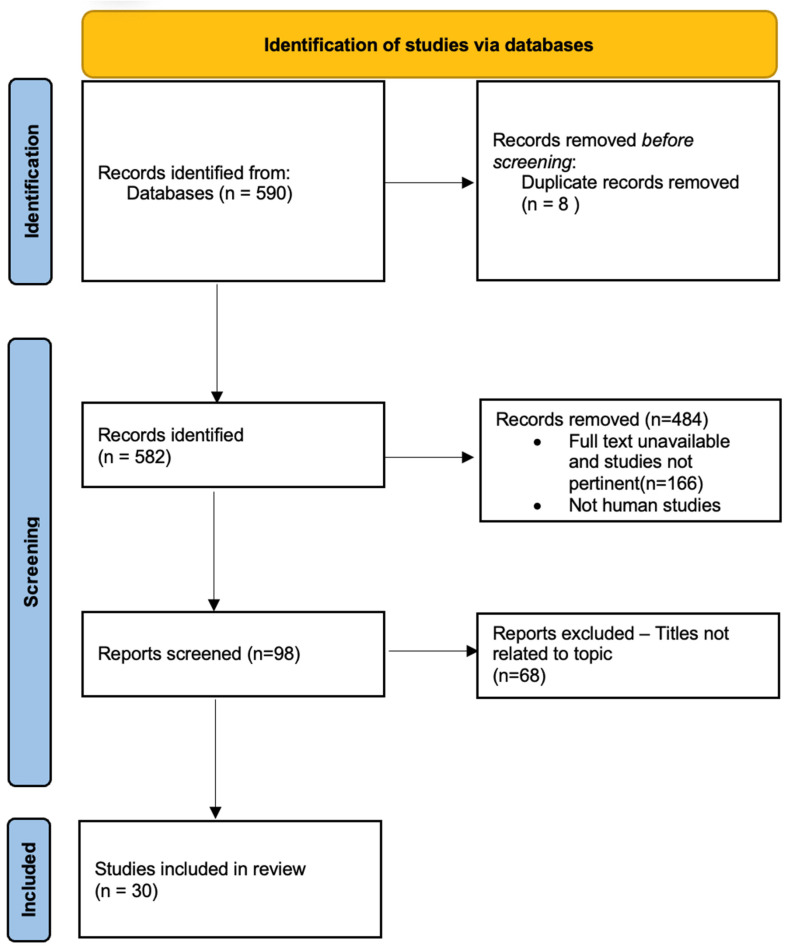
Material and methods. Identification of studies via databases. PubMed search with 590 articles. Duplicate articles and animal studies were removed. All articles with titles not related to the topic were also removed. After a close review of the abstracts by two blinded investigators, 25 articles were retained for a final review.

The initial search terms included were “hydrogenated water”, “hydrogen water”, “hydrogen-rich water”, “molecular hydrogen”, “hydrogenated water”, “antioxidant”, “anti-inflammatory”, “anti-apoptotic”, “fatigue”, “oxidative stress”, and “cytoprotective”. This PubMed search yielded a total of 590 articles. Duplicate articles and animal studies were removed. All articles with titles not related to the topic were eliminated. After reviewing the abstracts by two blinded investigators (RD and RM), 30 articles were retained for a final review (Figure 1). Our inclusion criteria were human studies with hydrogen-rich water and comparison groups or pertinent clinical or pathophysiological information in cohort studies, case-control studies, clinical trials, or observational studies. We excluded opinion articles, editorials, and book chapters for this systematic review. We also excluded results on the therapeutic effects of hydrogen gas inhalation and the injection of hydrogen-rich saline, and only included hydrogen-rich water studies.

Studies were exported from PubMed to Rayyan software (https://www.rayyan.ai/). Two investigators (PS and GD) screened titles and abstracts independently to select appropriate studies. Afterward, the investigators (GD and HG) assessed the full texts of the articles to determine final eligibility. Conflicts were discussed with a non-reviewing investigator (RK) and were resolved. The study was also registered on PROSPERO (CRD42023445460). The final review was conducted with 30 articles (Figure 1).

One of the first documented human studies on hydrogen-rich water was conducted in 2008. An experimental drink was produced by dissolving hydrogen gas into water under high pressure. It was used for patients with type 2 diabetes or impaired glucose intolerance. Common medical disorders like hypertension, diabetes, and atherosclerosis are associated with oxidative stress. Although the sample size was small, drinking hydrogen-rich water did have some benefits in preventing type 2 diabetes mellitus [5]. Hydrogen-rich water can be consumed orally and can be produced in multiple ways, which include hydrogen-generating tablets, infusion machines, water generators, and ionizers. The effective delivery of hydrogen through inhalation might be difficult. An advantage of using hydrogen-rich water to deliver molecular hydrogen is that it can be easily administered and is portable [6]. The beneficial effects can be seen even at low concentrations [6].

**Figure 2:**
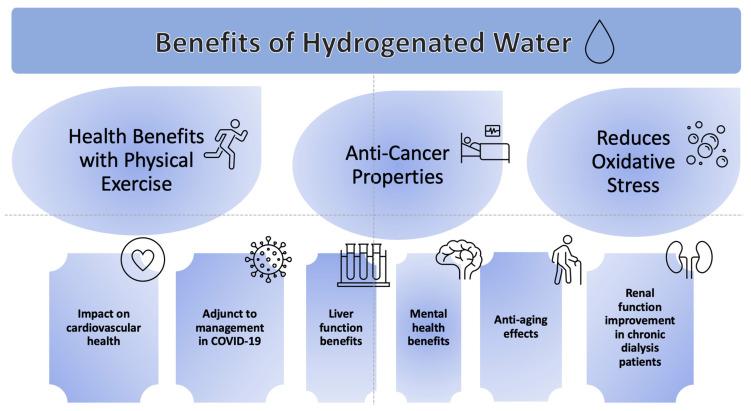
Summary of benefits of hydrogen-rich water.

We have divided the summary of our findings into the following subheadings (Figure 2).

### 2.1. Health Benefits of Hydrogen-Rich Water with Physical Exercise

Physical activity is good for several reasons, offering numerous mental, emotional, and physical benefits [7]. Studies have also been done to see the effect of physical activity on mental health [7]. Some advocates of hydrogen-rich water believe that it has the potential to provide multiple health benefits with physical exercise, like enhanced performance and recovery [8]. Although the data are still limited and inconclusive, studies have shown encouraging results, as discussed below.

Physical exercise can result in increased reactive oxygen species, which can cause damage to tissue and fatigue. With most forms of exercise, sensations of fatigue and exhaustion occur after some time. Research has shown that drinking hydrogen-rich water before exercising can mitigate the effects of fatigue and build endurance [8]. A study conducted on cyclists showed that a 7-day consumption of nano-bubble hydrogen-rich water improved the anaerobic performance of trained cyclists compared to that of untrained ones [9]. There is a build-up of lactic acid in the muscles with exercise. Hydrogen-rich water administered pre-workout showed decreased blood lactic acid levels at a higher intensity and improved ventilatory efficiency [10]. Pre-workout hydrogen-rich water has also been gaining traction. The supplementation of hydrogen-rich water prior to exercise in other studies has been shown to reduce fatigue as well as improved endurance in the later stages of repeated sprints [11].

Not all studies have demonstrated encouraging results. A randomized, double-blind, placebo-controlled crossover design study by Botek et al. [12] showed unclear effects on fatigue. Study participants were placed in either placebo or hydrogen-rich water groups. Interestingly, hydrogen-rich water had an unclear effect on race time and minimal impact on heart rate. Endurance performance was improved by 1.3% in the slowest runners with pre-race hydration with 1680 mL hydrogen-rich water, but the effect on the fastest runners was unclear as there was 0.8% deterioration. Also, in the slowest runners, there was an improvement in race heart rate by 3.8%, along with an improvement in performance; however, in the fastest runners, the change was unclear (0.1%). Depending on the running ability of individuals, the effect of hydrogen-rich water on performance can vary [12].

Training and competition are part of athletes’ lives. Oxidative stress has a vital role in the development of inflammation [3]. A study was undertaken on female juvenile soccer players from Suzhou, China, with the consumption of hydrogen-rich water for 2 months in the treatment group, which showed changes in serum malondialdehyde, interleukin-1, interleukin-6, and tumor necrosis factor-α (TNF-α) levels, with an increase in serum superoxide dismutase and total antioxidant capacity levels [13]. After 8 weeks, serum malondialdehyde levels decreased from 13.80 ± 3.33 to 12.69 ± 1.94 μM in the hydrogen-rich water group and from 16.67 ± 4.19 to 15.79 ± 3.07 μM in the control group (*p* = 0.000). In the same period, the interleukin-1 levels went up from 29.32 ± 7.09 μM to 34.47 ± 6.22 μM in the hydrogen-rich water group and from 32.56 ± 7.61 to 42.94 ± 6.24 μM in the control group (*p* = 0.002) [13]. The levels of interleukin-6 increased from 8.74 ± 2.57 to 12.37 ± 3.2 ng/L in the hydrogen-rich water group and from 10.53 ± 1.62 ng/L to 24.88 ± 6.11 ng/L in the hydrogen-rich water group after 8 weeks (*p* = 0.000). The serum TNF-α levels increased from 49.46 ± 11.59 to 107.00 ± 13.89 μM in the hydrogen-rich water group and from 60.57 ± 10.09 to 132.24 ± 10.46 μM in the other group (*p* = 0.000). For superoxide dismutase, the levels decreased from 14.07 ± 1.91 to 13.69 ± 2.10 U/mL in the hydrogen-rich water group, while it decreased from 13.14 ± 2.18 to 13.01 ± 1.08 U/mL in the control group (*p* = 0.027) [13].

Studies have shown the antioxidant, anti-apoptotic, cytoprotective, and anti-inflammatory properties that hydrogen can exert on the cell. Hydrogen-rich water has the potential to be used for the treatment of many diseases, including cardiovascular and neurodegenerative diseases, among others [14].

Hydrogen-rich water can improve acidosis due to exercise, energy levels, and enhanced muscular performance in athletes [15].

### 2.2. Impact of Hydrogen-Rich Water on Oxidative Stress

Oxidative stress is known to be a common cause of lifestyle-related diseases, the aging process, and even cancer [4]. Reactive oxygen species are generated internally as we breathe and consume oxygen [4]. Hydrogen is effective against oxidative stress and is also known for its anti-inflammatory [4] and anti-allergy [4] benefits. Hydrogen reduces the oxidative damage that occurs between biological molecules and hydroxyl radicals [1]. With this reduction in oxidized macromolecules, there is a decrease in cellular and mitochondrial injuries [1]. Another added advantage is that, even at higher concentrations, hydrogen has no cytotoxicity [4]. Also, in mixed deep diving gas, hydrogen gas in high concentrations is used for inhalation to prevent arterial gas thrombi and to prevent decompression sickness [4].

### 2.3. Impact of Hydrogen-Rich Water on Cardiovascular Health

The effects of molecular hydrogen on cardiovascular disease are interesting. Molecular hydrogen controls signal transduction and gene expression, suppressing pro-inflammatory cytokines and decreasing reactive oxygen species production. It also leads to the activation of the nuclear factor erythroid 2-related factor 2 (Nrf2) antioxidant transcription factor. Even though hydrogen has antioxidant, anti-inflammatory, and anti-apoptotic effects, the exact mechanism of action is poorly understood. There are data to suggest that the mild hormetic-like effects of hydrogen might be responsible for these benefits, but more research is still needed [1].

Hydrogen-rich water can help in the management of hyperlipidemia [16]. Twenty patients (10 smokers and 10 non-smokers) who received hydrogen-rich water for 10 weeks showed a drop in total cholesterol levels from 6.42 mM to 5.47 mM (*p* < 0.01), whereas LDL levels dropped only from 3.96 mM to 3.24 mM (*p* < 0.05). It is interesting to note that the beneficial effects were better in smokers than non-smokers. Additionally, there was no effect on the levels of HDL-C. The levels of serum triglyceride were decreased with hydrogen-rich water treatment in smokers from 2.93 mM to 2.3 mM, but the levels in non-smokers went from 1.49 mM to 1.67 mM [16].

Hydrogen-rich water can potentially decrease LDL-C and apoB levels while improving HDL function. It may also have a role in the prevention of metabolic syndrome [16]. In another study [17], 20 subjects were selected for an 8-week study. Patients with potential metabolic syndrome received hydrogen-rich water (1.5–2 L). There was a 39% increase in antioxidant superoxide dismutase (SOD) (*p* < 0.05) and a 43% decrease in thiobarbituric acid (TBARS) in urine (*p* < 0.05). Also, high-density lipoprotein (HDL) cholesterol increased by 8%. Fasting glucose levels were unchanged [17]. A randomized, double-blinded, placebo-controlled trial in 60 individuals with metabolic syndrome yielded encouraging results. Clinical baseline data was obtained at baseline after an observation period of 1 week. Then, subjects were randomized to high-concentration hydrogen-rich water (>5.5 millimoles of H_2_ per day) vs. the placebo group. The use of high-concentration hydrogen-rich water was shown to decrease blood glucose and cholesterol levels, improve serum hemoglobin A1c, and also to improve inflammatory biomarkers (*p* < 0.05). Interestingly, it also led to an improvement in waist-to-hip ratio and body mass index [18].

Furthermore, in unstable angina patients, the consumption of hydrogen-rich water with conventional medications was shown to relieve symptoms associated with that condition (60% vs. 90%, χ^2^ = 4.800, *p* < 0.05) [19]. The hydrogen-rich water group was noted to have lower total cholesterol (35% vs. 15%), apoB (40% vs. 15%), and LDL-C (40% vs. 20%) levels compared to the control group [18]. Hydrogen-rich water can also improve the endothelial function of the arteries to improve cardiovascular health [20]. In evaluating vascular endothelial function and cardiovascular disease, the reactive hyperemia index (RHI) using peripheral arterial tonometry (PAT) is useful. RHI improved by 25.4% (*p* < 0.05) after 2 weeks of hydrogen-rich water consumption [20].

### 2.4. COVID-19 and Hydrogen-Rich Water

The COVID-19 pandemic has significantly impacted our lives in the last few years [21]. Although it is not a health emergency globally today, it is important to be vigilant as new variants have emerged in the last few years [21]. It is interesting to note that, as hydrogen inhalation has anti-inflammatory, antioxidant, and anti-apoptotic action, it can aid in the management of COVID-19 [22]. The antioxidant and biological effects of hydrogen-rich water are seen even after hydrogen is cleared from the body [22]. Molecular hydrogen therapies were also seen to be effective in remediating the dangerous consequences of COVID-19 infection. Hydrogen administration inhibited cytokine cascade and decreased inhalation resistance in patients with mild to moderate disease [23]. Although hydrogen has shown potential in the last few years, it is still too early to conclude its usefulness.

### 2.5. Hydrogen-Rich Water and Dialysis

As we go forward, hydrogen-rich water has started to make an impact on various diseases and disorders. Oxidative stress plays an important role in chronic kidney disease pathology [24]. In chronic dialysis patients, a study showed that electrolyzed hydrogen-rich water (EHW) intake can improve blood urea nitrogen (BUN) and renal function. It can also decrease oxidative stress in patients with chronic dialysis during their hemodialysis sessions [24]. Also, in hemodialysis (HD) patients, fatigue is often attributed to oxidative stress. A study was done to see if hemodialysis solutions with electrolyzed hydrogen-rich water would affect autonomic function and fatigue. The use of HD solutions with electrolyzed hydrogen-rich water decreased fatigue in patients on both HD and even on HD-free days [25]. Alkaline-electrolyzed-reduced water (ERW) has been in use for many years. It has been proven that the primary agent responsible for oxidation reduction potential and the therapeutic effects of ERW was H_2_ [26].

### 2.6. Effect of Hydrogen-Rich Water on Cancer

As medical science continues to advance, molecular hydrogen has started to find its way into oncology. Colorectal cancer is a common cause of death due to cancer, and removal of tumors is still the mainstay of treatment [27]. Hydrogen-rich water did show anti-cancer properties in a study [27]. With its antioxidant properties and ability to decrease oxidative stress, it could be a potential game changer in the future. A combination of hydrogen-rich water and 5-fluorouracil (5-FU) did show improvement in the size of the tumor, fibrosis, and content of collagen [27]. Another systematic review was to see molecular hydrogen’s effect as an adjunctive therapy for cancer treatment. A total of 677 articles were reviewed, and 27 were selected for final review. Hydrogen was noted to have potential in treatment, overall prognosis, quality of life, and tumor reduction [28].

### 2.7. Benefits of Hydrogen-Rich Water on Mental Health

Mental health is another aspect of today’s world that cannot be ignored. As we move on from the COVID-19 pandemic, it is crucial to understand the effect it had on mental health. Higher rates of depression, anxiety, and stress were seen in the general population in many countries [29]. A study showed that subjects who drank hydrogen-rich water for 4 weeks had improved mood, anxiety, and overall mood [30]. Another interesting study was performed on women with panic disorder [31]. The control group was started on psychological treatment and a placebo, while the treatment group was placed on psychological treatment and 1500 mL of hydrogen-rich water daily for 3 months. Results showed no significant difference between the control and treatment groups; however, it should be noted that the treatment group did show a significant decrease in pro-inflammatory cytokines (IL-6, IL-1β, IL-12, and TNF-α) compared to the control group. In the treatment group, after treatment with hydrogen-rich water, IL-1β levels decreased from 94.1 to 65.5, IL-12 from 75.75 to 54.5, IL-6 from 72.3 to 51.67, and TNF-α from 74.5 to 49.25 (all data with *p* < 0.05). This may have led to an improvement in physical health and body pain [31].

### 2.8. Hyroden-Rich Water and Liver Function Benefits

As hydrogen-rich water decreases oxidative stress, a study was done on patients with chronic hepatitis B. Hepatitis B is a global health problem and can be life-threatening. Subjects were administered hydrogen-rich water (1200–1800 mL/day, twice daily), which improved liver function and reduced HBV DNA [32]. It also decreased oxidative stress in chronic hepatitis B patients [32]. Non-alcoholic fatty liver disease (NAFLD) affects 25% of the population. Liver dysfunction can be caused by inflammation, oxidative stress, and aberrant cellular signaling. It has been shown that the administration of hydrogen-rich water can have beneficial effects for these patients [33]. Thirty individuals with NAFLD were administered hydrogen-rich water in a randomized, double-blind, placebo-controlled study for 8 weeks. Decreased body mass index and weight (≈1 kg) were observed in the treatment group [33]. As treatment for NAFLD remains elusive, a few studies have been done to assess the benefits of hydrogen-rich water on the disease. Hydrogen-rich water was shown to decrease fat accumulation in the liver and has the potential to be used as an adjuvant treatment for mild to moderate NAFLD [34].

### 2.9. Effect of Hydrogen-Rich Water on Aging

The risk factor for many cardiovascular diseases, neurodegenerative disorders, and even cancer is age [35]. With hydrogen-rich water making news in the last few years, a study was undertaken to assess the effects of hydrogen-rich water in men and women above the age of 70 and whether it affected aging. It was found that drinking hydrogen-rich water for 6 months was harmless and also had a favorable effect on many of the factors associated with aging, like pain, metabolic processes in the brain, strength in the lower extremities, etc. [35]. Another study showed the hydrogen has anti-aging effects through the (Nrf2) pathway on vascular endothelial cells. Therefore, it has the potential to increase longevity. This can even be seen after temporary exposure to hydrogen [36].

## 3. Results and Discussion

Hydrogen-rich water has gained worldwide attention over the last few years given its potential health benefits. Hydrogen-rich water’s effect on exercise capacity and physical endurance is of particular interest to individuals with a fondness for physical activity. Additionally, the potential for a positive impact on cardiovascular function can reduce the risk of heart disease. Additionally, the possible effect of hydrogen-rich water on mental health is intriguing, with the initial results being encouraging. Also, its effect on anti-cancer properties holds promise in the field of oncology. Given its potential to positively impact liver function, anti-aging, and oxidative stress, hydrogen-rich water is a subject of ongoing research and growing interest. Hydrogen-rich water offers several potential strengths, including its antioxidant, anti-inflammatory, and anti-apoptotic properties. It can also help decrease oxidative stress. Some studies showed that it may also improve physical endurance, cognitive function, and overall well-being. Moreover, hydrogen-rich water is mostly considered safe, with no to minimal side effects. There is growing interest in the benefits of hydrogen-rich water, and it may also have potential applications in medical therapies.

Hydrogen-rich water can aid in the excretion of toxins from the liver to the bile and promote their fecal excretion by enhancing the efflux pumps of Mrp2 and *p*-glycoprotein. In a study [37], there was no effect on plasma mineral ions with a small change in the concentrations of calcium, magnesium, and sulfate between the hydrogen-rich water and control water groups. Interestingly, the hydrogen-rich water group had a higher volume of water intake as compared to the control group, with regular water consumption (81.8 ± 5.1 mL/day in the hydrogen-rich water group compared with 73.9 ± 5.0 mL/day in the control group, *p* < 0.05). This might have been due to better palatability with the hydrogen-rich water group. Magnesium intake has been shown to decrease cardiovascular and cerebrovascular disease mortality in human beings [37]. In study [37], hydrogen-rich water had a higher concentration of magnesium than the control group (22.8 ppm in the hydrogen-rich water group compared to 10.2 in the control group). Magnesium was also shown to decrease levels of blood glucose in rat liver by interfering with the gluconeogenesis pathway. This may have led to a decrease in plasma glucose levels of 7.7% (*p* < 0.05) in the hydrogen-rich water group compared to the control group [37].

Comparison of hydrogen-rich water with other health supplements, such as protein powder, herbal supplements, collagen, and vitamins, is challenging yet essential, as they serve different purposes and can affect health and well-being.

Over the last few decades, protein powder has become popular among individuals with an interest in physical activity looking to support their fitness goals. There have been studies undertaken to assess the impact of protein powders on physical endurance and fitness. In healthy individuals undergoing chronic endurance training, protein supplements were shown to increase aerobic capacity further, improve time trial performance, and lead to lean mass gain [38]. Another study showed that protein supplements and carbohydrate strategies in individuals undergoing endurance exercise can decrease muscle damage but did not improve endurance capacity [39]. High protein intake for prolonged periods has been linked to various health concerns, including increased risk of renal disorders, calcium metabolism, the progression of coronary artery disease, and even cancer [40]. There are not much data available specifically comparing protein powder and hydrogen-rich water strategies for individuals engaging in physical activity.

A separate study was done on 89 individuals to see the effect of protein powder (on whey or casein protein for 12 weeks of consumption) on cholesterol levels [41]. It caused decreased total cholesterol levels by 7% in the whey protein group compared to the baseline and a 9% decrease in the whey protein group compared to the casein group. LDL levels were also decreased by 7% in the whey group compared to the baseline. Protein powder and hydrogen-rich water can both be a part of a dietary regimen to support fitness goals. While hydrogen-rich water provides potential antioxidant and anti-inflammatory effects [15], protein supplementation is used for lean muscle gain and increased aerobic capacity. As medical science continues to evolve, we might better understand how these two strategies can be used synergistically or in certain scenarios.

Herbal supplements are commonly used in different parts of the world. A few studies were done to evaluate the impact of herbal supplements on COVID-19 patients. Zinc sulfate could decrease the duration of olfactory dysfunction. However, more well-designed studies are needed in the future given the low quality of included trials [42]. Also, there has been a debate on using herbal supplements to treat mood disorders. A few are effective in the management of depression, like *Catha edulis, Tinospora cordifolia, Curcuma longa, Rhodio larosea, Crocus sativus,* etc. [43]. There has also been evidence in favour of the use of *Passiflora spp. (passionflower)* and *Piper methysticum (Kava)* in treating anxiety, and *Crocus sativus (saffron*) and *Hypericum perforatum (St John’s wort)* for treating depression. In schizophrenia, *Ginkgo biloba (ginkgo)* has been used as an adjunctive treatment [44]. EGb 761, a special extract of *Gingko biloba*, stabilizes mood and improves cognitive functioning in elderly individuals with cognitive impairment [45]. In this study, 176 patients with generalized anxiety disorder or adjustment disorders with anxious mood were randomized to one of the three groups for 4 weeks: 480 mg EGb 761, 240 mg EGb 761, or placebo. The primary outcome measure used was the Hamilton rating scale for anxiety (HAMA). In the high-dose EGb 761 group, the HAMA score decreased by −14.3, −12.1 in the low-dose EGb 761 group, and by −7.8 in the placebo group (*p* = 0.0003 in the high-dose group and *p* = 0.01 in the low-dose group) [45].

Going forward, there needs to be more focus on quality research to establish herbal supplements’ efficacy and safety as they are not as well established as the psychotropic medications currently in use.

Collagen is associated with skin health and overall well-being. It constitutes approximately 80% of the dry weight of skin [46]. With aging, there is a decrease in the enzymes involved in its processing that, in turn, decreases the fibroblasts involved in the synthesis of collagen [46]. Topical and oral collagen can reduce skin aging [47]. The effects of vitamins and nutrients on aging are also shown [46]. Supplementation with zinc, carotenoids, selenium, and vitamins C and E could slow aging [48].

Hydrogen-rich water, protein powder, herbal supplements, and vitamins, etc., are distinct dietary supplements and have different effects on the body. There are not much data available comparing hydrogen-rich water to protein powder, herbal supplements, collagen, and vitamins. 

Many factors affect the therapeutic effect of hydrogen-rich water, such as the hydrogen concentration in water, hydrogenation methods, and best duration, etc. This, in turn, can lead to different results. As the hydrogen concentration and quality can vary in studies, it can be challenging to compare results. Although the results of many studies reviewed have been encouraging, it should be noted that many were conducted in animals [2], and some used small sample sizes [48]. This can have an impact on the statistical power of the research and the generalizability of findings. Research trials with a large sample size would be needed in the future. We also noticed that the studies on hydrogen-rich water primarily focused on short-term benefits [48] and did not consider the long-term effects. Some studies [16] did not have a placebo control group, so it is difficult to determine whether the results could be attributed to hydrogen-rich water.

Also, it should be noted that, as some of the studies might have been supported by organizations with an interest in hydrogen-rich water products, there could be commercial biases in publication. A proper conflict of interest analysis is required as we move forward. Over the last few years, there has been a better understanding of the effects of hydrogen, with studies showing that the primary molecular target of hydrogen is Fe-porphyrin [49]. The main target of hydrogen intracellularly is mitochondria, where oxidized Fe-porphyrin has been shown to be responsible for hydrogen’s destruction of reactive oxygen species. Fe-porphyrin has also been shown to rectify electron flow in disordered states. Quantum biology going forward can help us better understand the exact mechanism of molecular hydrogen on mitochondria [50]. Hydrogen-rich water also leads to the activation of Nrf2, which has been shown to have a positive impact on cardiovascular health [1] and anti-aging effects [35]. We should look forward to developing therapeutic protocols and validating the potential of hydrogen-rich water in a clinical setting.

## 4. Conclusions

Increased interest and continuous study are being directed toward the possible health advantages of hydrogen-rich water in a variety of areas, including physical endurance, exercise capacity, cardiovascular disease, liver function, COVID-19, mental health, anti-aging research, and oxidative stress. These potential consequences have aroused debate in the scientific and medical industries. Even though there is great potential in understanding the benefits of hydrogen-rich water, we still have to overcome the existing limitations. We need well-designed studies in humans, with large sample sizes and long-term trials, to ascertain the benefits.

## Data Availability

No new data were created.
